# Clinical research progress of callisperes^®^ of drug-loaded microsphere arterial chemoembolisation in the treatment of solid tumors

**DOI:** 10.1007/s12672-024-01030-z

**Published:** 2024-05-13

**Authors:** Qin Wang, Lujian Zhu, Qiyue Sheng

**Affiliations:** Department of Infectious Diseases, Affiliated Jinhua Hospital, Zhejiang University School of Medicine, Jinhua, China

**Keywords:** CalliSperes^®^ of drug-loaded microsphere, Arterial chemoembolization, Treatment, Solid tumors, Research progress

## Abstract

The incidence and mortality of cancer is ever-increasing, which poses a significant challengesto human health and a substantial economic burden to patients. At present, chemotherapy is still a primary treatment for various cancers. However, chemotherapy kills tumors but also induces the related side effects, whichadversely impacting patient quality of life and exacerbating suffering. Therefore, there is an urgent need for new and effective treatments that can control tumor growth while reducing the side effects for patients. Arterial chemoembolization has been attracted much attentionwhich attributed to the advantage of ability to embolize tumor vessels to block blood and nutrition supplies. Thus, to achieve local tumor control, it has become an effective means of local tumor control and has been widely used in clinical practice. Despite its efficacy, conventional arterial chemoembolization techniques, limited by embolization materials, have been associated with incomplete embolization and suboptimal drug delivery outcomes. Gradually, researchers have shifted their attention to a new type of embolic material called CalliSperes^®^ drug-eluting embolic bead (DEB). DEB can not only load high doses of drugs, but also has strong sustained drug release ability and good biocompatibility. The integration of DEBs with traditional arterial chemoembolization (DEB-TACE) promises targeted vascular embolization, mitigated tumor ischemia and hypoxia, and direct intravascular chemotherapy delivery. It can prevent cancer cell differentiation and accelerate their death, meanwhile, directly injecting chemotherapy drugs into the target blood vessels reduced the blood concentration of the whole body, thus reduced the toxic and side effects of chemotherapy. Furthermore, DEB-TACE's sustained drug release capability elevates local drug concentrations at the tumor site, amplifying its antitumor efficacy. Therefore, DEB-TACE has become a hot spot in clinical research worldwide. This review introduces the pathogenesis of solid tumors, the background of research and biological characteristics of DEB, and the action mechanism of DEB-TACE, as well as its clinical research in various solid tumors and future prospects. This review aims to provide new ideas for the treatment of DEB-TACE in various solid tumors.

## Introduction

According to the global incidence rate of cancer in 2020 released by the International Agency for Research on Cancer, one of the leading causes of human death is malignant tumors [1]. In 2020, the ammount of new cancer cases worldwide is estimated to reach 19.3 million, alongside nearly 10 million fatalities. Notably, China’s malignant tumor incidence ranked second globally. It is estimated that there will be a 57.5% increase in 2040 compared to that in 2020, and the most common malignant tumors are solid tumors such as lung cancer, colorectal cancer, liver cancer, and gastric cancer [2, 3]. Conventional chemotherapy remains the principal treatment modality for these malignancies [4–6]. However, the efficacy of traditional systemic chemotherapy is often compromised by low drug concentrations at tumor sites, resulting in suboptimal therapeutic outcomes. While killing tumors, it also damages normal tissues and cells in the human body, which brings toxic side effects to patients, exacerbates patient suffering, and diminishes their quality of life [7]. With the update of new technologies and concepts, it is currently a top priority in clinical research to further improve the efficacy of chemotherapy drugs, reduce toxic and side effects, and improve the precision of tumor treatment [8]. Research has shown that CalliSperes^®^ drug-eluting embolic bead (DEB) combined with arterial chemoembolisation (DEB-TACE) can embolize tumor target blood vessels, which not only allows chemotherapy drugs to be injected into the target blood vessels, and thus reduce systemic blood drug concentration and alleviate the toxic side effects of chemotherapy, but also causes tumor ischemia and hypoxia, inhibits the nutrient absorption by cancer cells, prevents their differentiation, and promotes their death. Additionally, DEB-TACE can continuously release chemotherapy drugs, which can increase the local drug concentration around tumors and enhance the anti-tumor effects [9]. Therefore, DEB-TACE has become a hot topic in clinical research. This review summarized the therapeutic background, mechanism of action, clinical research, and prospects of DEB-TACE on solid tumors.

## Concept and growth mechanism of solid tumors

### Concept of solid tumors

In clinical medical diagnosis and treatment, tumors can be categorized into solid and non-solid subtypes based on the tissue origin, morphological differences, and growth rate [10–12]. Solid tumors can be continuously proliferated and differentiated through tumor cells, eventually forming visible masses under imaging examinations [13, 14]. However, non-solid tumors cannot be observed through gross visualizationand they mainly manifest in the blood system. Non-solid tumors can only be diagnosed through relevant laboratory tests [15]. Therefore, solid tumors and non-solid tumors are different. The treatment of the former primarily includes surgery, radiotherapy and chemotherapy, targeted therapy, and immune checkpoint inhibitors [16, 17]. Whereas non-solid tumors are predominantly managed with pharmacological treatments [18].

### Growth mechanism of solid tumors

To improve the therapeutic effects on solid tumors, we need to clarify the growth mechanism of solid tumors. The growth mechanism of solid tumors is complicated, and the pathogenesis includes the following points. (1) The first is oncogenic driver genes: Mutations, deletions, or rearrangements within specific genes [19] result in abnormal activation of tumor gene functions, promoting the continuous growth and reproduction of tumors [20–22]. Genetic testing technology has identified some driver genes such as EGFR, KRAS, and ALK [23]. (2) The second is immune escape: Tumor cells manipulate immune checkpoints to evade detection and destruction by the host’s immune system, employing mechanisms that deactivate T cells and dendritic cells. This immunological stealth allows early-stage tumors to persist and expand undetected [18]. The third is abnormal glucose metabolism: Solid tumors preferentially engage in anaerobic glycolysis, producing substantial lactate and engendering an acidic microenvironment [24]. This adaptation not only facilitates vascular neoformation in carcinoma but also enhances tumor aggressiveness and recurrence risk [25]. The fourth is abnormal lipid metabolism:It characterized by the accumulation of lipid droplets in mutated tumor cells, supports the viability and proliferation of cancer stem cells. These lipid reserves contribute to cellular immune responses and the synthesis of prostaglandin-2, promoting further differentiation, growth, and metastasis of cancer cells [26–28]. The last factoris the abundant blood supply to carcinoma: the normal tissues of the human body excrete carbon dioxide and waste materials, ingest oxygen and nutrients, and complete the metabolism of tissue cells through the blood vessels. A previous study has proved that solid tumors have an independent vascular system, and tumor cells stimulate the growth of vascular factors to promote the continuous establishment of blood vessels [29], which becomes a bridge for transporting nutrients and excreting metabolites. Additionally, solid tumors coexist with the host blood vessels, stolen oxygen and nutrition from the host's original tissue, and colonize the normal tissue to grow [30]. These mechanisms show the complexity of solid tumor pathogenesis and highlight the necessity for targeted therapeutic strategies to inhibit tumor growth and progression.

## Callisperes^®^ of drug-loaded microsphere

### Research background of callispheres^®^ drug-carrying microspheres

The biotechnological innovations has catalyzed the development of novel chemical materials in the medical arena, among which DEB represent a pivotal advancement in clinical embolotherapy applications [31]. In the 1970s, researchers produced embolic materials, which were divided into solid and liquid parts in the early years, based on their physical states and early application. In contrast, solid embolic materials included permanent and reabsorbable materials according to the differences in biological properties of materials [32]. The permanent mainly included stainless steel particles, polyvinyl alcohol (PVA), and sodium alginate microspheres [33–35], while the reabsorbable materials was primarily a gelatin sponge [36]. Liquid embolization materials are mainly iodine oil and anhydrous ethanol. Both forms, however, were associated with potential complications, including inflammatory reactions, incomplete embolization, and toxic by-product release. Addressing these challenges, the drug-loaded microspheres have been developed, such as foreign DC/LC beads, HepaSpheres, Embosphere, and Embozene microspheres. Their clinical efficacy and safety have been confirmed by clinical trials. The new DEB has been approved for clinical treatment and research in 2015 [37–39].

### Biological characteristics of callispheres^®^ drug-carrying microspheres

DEB has been widely used in clinics because of its unique biological characteristics. DEB is a component substructure independently developed based on traditional PVA [9]. It carries an anionic sulfonic acid group and cationic amino group of chemotherapy drugs for exchange to complete drug loading. Studies have shown that DEB can accelerate the loading of drugs in a 5% glucose solution [40–42]. DEB can be divided into five types according to the diameter: 100 ~ 300, 300 ~ 500, 500 ~ 700, 700 ~ 900, and 900 ~ 1200 μm. DEB has the following characteristics. (1) Good elasticity: DEB with a diameter of 300 ~ 500 μm would be compressed to 1/2 of the original volume after drug loading, and its diameter would return to the original size after drug release. (2) Sustained releasing force: the study has found that DEB's drug release could reach 80% of the total cure in the first 12 h, and the remaining 20% maintains a slow release for up to 1 month. The concentration and speed of drug release of microspheres with different diameters are different, and the maximum drug release can reach 100% [43]. (3) Biocompatibility: even if a large amount of DEB stays in blood vessels, it will not damage vascular endothelial cells and vascular walls or cause inflammation and rejection with blood vessels. Additionally, when mixed with iodine-containing contrast media to form a suspension, the drug leakage rate is less than 1%. (4) Non-clogging catheter: since PVA has the component of chitosan film, DEB exists in the form of multiple pores before drug loading [44–46], and its surface structure is smooth after drug loading. Therefore, it is not easy to combine with coagulation molecules in the blood and lead to vascular embolism. At the same time, considering that the specific gravity of human blood is about 1.05 [47], and the particular gravity of DEB before and after drug loading fluctuates between 1.04 and 1.07, both DEB before and after drug loading can float in the blood vessels [48]. According to DEB's biological characteristics, it can be seen that DEB can load high-dose drugs, with strong sustained release ability and good biocompatibility, which provides a solid foundation for the subsequent application of DEB-TACE.

### Mechanism of callisperes^®^ drug-eluting bead transarterial chemoembolization

DEB-TACE is optimized based on Conventional transarterial chemoembolisation (c-TACE). The mechanism of c-TACE is to insert the catheter into the blood vessel to establish a channel through superselection technology [49], followed by the injection of the mixed liquid of iodide and chemotherapy drugs into the artery, and then the injection of the embolic agent to achieve vascular embolization, thereby achieving an anti-tumor effect [50]. However, c-TACE's limitations include incomplete embolization and potential collateral circulation, hindering complete tumor necrosis [51].

DEB-TACE is to use a new type of DEB mixed with fat-soluble chemotherapeutic drugs [52]. DEB-TACE integrates DEB with lipophilic chemotherapeutic agents, creating a robust hydrogen bond for drug attachment. DEB is spherical in appearance. After drug loading, its diameter is compressed to half of the original. After that, it enters the vascular microenvironment and begins to release the drug under the action of the ionic structure [53]. At the same time, the volume of DEB gradually increases, and the enlarged microsphere is embedded in the blood vessel to achieve complete embolization [54]. When the blood vessel is embolized, the blood supply is blocked, which reduces tumor tissue perfusion, cuts off the nutrient source of tumor tissue, causes hypoxia and ischemia of tumor tissue, and inhibits tumor cell growth and differentiation, thereby resulting in tumor cell necrosis to achieve tumor shrinkage or remission. DEB-TACE also avoids the metabolism of drugs through the blood circulatory system, and the concentration of drugs reaching the target blood vessels is still at a high level, which increases the distribution density and concentration of anticancer drugs in the tumor. In contrast, the concentration of drugs in the plasma is decreased. For this reason, the anti-tumor efficacy would be increased, and the toxic side effects caused by chemotherapy drugs would be alleviated.

## Research progress of callisperes^®^ drug-eluting bead transarterial chemoembolisation in solid tumors

Given the vascular-dependent growth characteristic of solid tumors, DEB-TACE offers a targeted approach for local chemoembolization, addressing tumor vasculature directly. This section reviews the clinical application of DEB-TACE across various solid tumors.

### Clinical application of callisperes^®^ drug-eluting bead transarterial chemoembolisation in hepatocellular carcinoma

Primary liver cancer ranks sixth in the global incidence rate of cancer. The incidence rate of liver cancer in China is ever-increasing year by year. According to the statistics of the worldwide incidence rate of cancer in 2020, its mortality has risen to second place [55]. As China is a big country with chronic viral hepatitis B, half of the world’s liver cancer patients are present in China; the preferred treatment for early liver cancer is surgical resection. However, in the clinical diagnosis and treatment, most liver cancer patients are diagnosed at an advanced stage. According to the anatomical and physiological characteristics of the liver, the hepatic artery is the blood supply to the liver 1/4–1/3. Therefore, blocking the hepatic artery may induce liver cancer tissue ischemia and necrosis [56]. At present, c-TACE has been widely used in patients with liver cancer. On this basis, DEB-TACE has been gradually used in patients with advanced liver cancer. In an animal experiment, 60 rabbits were randomly grouped: high-dose DEB-TACE group, low-dose DEB-TACE group, c-TACE group, Hepatic arterial infusion therapy (HAIC) group, and intravenous chemotherapy group. Each group was treated with 4 mg adriamycin, the low-dose DEB-TACE group was added with 4 mg epirubicin, and the high-dose DEB-TACE group was added with 8 mg epirubicin. The plasma concentration of adriamycin in each group was detected after treatment [57]. It found that drug concentration around the tumor tissue of rabbits in the intravenous chemotherapy group, HAIC group, and c-TACE group is lower than that in the DEB-TACE group [58]. At the same time, the degree of tumor necrosis in two DEB-TACE treatment groups (especially in the high-dose DEB-TACE group) is significantly higher than that in the other groups. This study showed that DEB-TACE played a more vital role in promoting tumor necrosis than traditional intravenous chemotherapy, HAIC, and c-TACE. A multicentre retrospective cohort study on DEB-TACE or c-TACE treatment of liver cancer patients has shown that the overall survival rate and partial response rate of the experimental group after three months of treatment are significantly higher than those of the control group and the disease control rate of the experimental group after one month. Three months of treatment was also higher than that of the control group, with statistical differences [59]. All the above results indicated that DEB-TACE is more advantageous than c-TACE in treating liver cancer. This result may be attributed to DEB’s unique biological characteristics. Namely, it can completely embolize the blood supply vessels of tumors, increase the time of chemotherapy drugs acting on tumors, and enhance the anti-tumor efficacy. To further clarify the effectiveness of DEB-TACE on liver cancer treatment, a prospective study has been jointly conducted by 24 medical institutions. According to whether the patients with liver cancer received c-TACE treatment before treatment and the number of times they received it, the patients are divided into three groups to receive DEB-TACE treatment respectively. Long-term follow-up has shown that DEB-TACE exhibits similar efficacy and safety in patients with different stages of liver cancer [60]. DEB-TACE is an effective treatment for advanced liver cancer patients who progress after other treatments. Due to the rich blood supply of the liver, the liver is prone to not only primary liver cancer but also secondary malignant metastasis fromother malignant tumors. DEB-TACE is effective on both primary and secondary liver cancer. A previous study found that the median overall survival of patients with colorectal cancer and liver metastases is up to 25 months if the clinician selects DEB-TACE for treatment [61]. DEB-TACE provides a new treatment plan and brings new hope for patients with metastatic liver cancer. In addition, to further inhibit the growth of residual tumors after surgery, Song Liu et al. have proposed to combine DEB-TACE with the targeted drug sorafenib in treating advanced large liver cancer. The results show that the median overall survival time of patients with advanced large liver cancer is 18.6 months, which is significantly longer than the median overall survival time of 12.7 months with DEB-TACE treatment alone [62]. The above studies have indicated the potential of DEB-TACE. The simple application of DEB-TACE can prolong the survival time of patients, improve the prognosis, and further extend the overall survival time of patients through a combination of targeted drugs. Therefore, the effectiveness of DEB-TACE combined with other anti-tumor drugs in treating tumors deserves further exploration.

### Clinical application of callisperes^®^ drug-eluting bead transarterial chemoembolisation in lung cancer

Lung cancer is one of the most common cancers worldwide [55]. According to the data analysis, it is estimated that lung cancer incidence will double by the end of 2030 compared with 2015 [63]. The blood supply of the lung comprises a pulmonary artery, pulmonary vein, pulmonary capillary network, and bronchial artery and bronchial vein [64]. Since most lung cancer grows in the bronchial epithelium [65], the bronchial artery serves as the main blood supply of lung cancer. These physiological and anatomical characteristics lay the foundation for bronchial artery chemoembolisation (BACE) of lung cancer. With the update of biomaterials, CalliSperes^®^ Drug-eluting bead bronchial artery chemoembolisation (DEB-BACE) has been gradually used in patients with unresectable advanced lung cancer. In a prospective study of Bin Shang et al. [66], 60 patients with stage II-IV lung cancer are enrolled: 30 patients with DEB-BACE treatment as the experimental group and 30 patients with bronchial arterial infusion chemotherapy as the control group. As shown by the results, the progression-free survival and overall survival after 3 and 6 months of treatment of the experimental group are significantly longer than that of the control group. However, the progression-free and overall survival after 6 months of treatment are statistically significant. Meanwhile, the concentrations of tumor marker carcinoembryonic antigen (CEA) and a soluble fragment of cytokeratin 19 (CYFRA21-1) are significantly lower in the peripheral blood of the experimental group than those of the control group. Additionally, studies have demonstrated that DEB-BACE has good short-term efficacy and safety in treating advanced non-small cell lung cancer and can significantly relieve the accompanying symptoms (such as hemoptysis, cough, and expectoration) [67–69]. Nevertheless, the above studies are all exploratory analyses aimed at advanced non-small cell lung cancer. To determine whether DEB-BACE can benefit patients with small-cell lung cancer, James Buxbaum et al. [70] have treated advanced small cell lung cancer with DEB-BACE. As shown by the results, 2 months and 6 months after surgery, neuron-specific enolase (NSE) and gastrin-releasing peptide precursors (proGRP) levels in advanced small cell lung cancer patients are decreased compared with those before, accompanied by the significantly improved postoperative quality of life; the median progression-free and survival are 5.1 and 9.0 months, respectively. In the study of He et al. [71], the median overall survival of patients with advanced small-cell lung cancer with second-line systemic chemotherapy is 7.7 months. Therefore, these two studies suggest that DEB-BACE is more advantageous than second-line chemotherapy for advanced small-cell lung cancer. The studies above have confirmed that DEB-BACE is effective on the treatment of lung cancer, which can not only alleviate the accompanying symptoms of tumors but also prolong the survival time of patients, reduce the concentration of serum tumor markers, and improve the prognosis. However, most studies above are single-arm experiments and the controlled studies are lacked. Therefore, cohort studies are needed in the future to further verify the efficacy of DEB-BACE.

### Clinical application of callisperes^®^ drug-eluting bead transarterial chemoembolisation in other solid tumors

DEB-TACE has the advantage of reducing bleeding, reducing tumor size, and preserving the essential physiological functions of the original organs [72]. With these advantages, DEB-TACE has been used to treat other types of solid tumors. Recently, Sinem Yaprak Karavana et al. have proposed microspheres as a new approach in treating bladder cancer patients [73]. This study suggests that for patients undergoing tumor resection, preoperative DEB-TACE treatment can block blood vessels to reduce hemorrhage risks, as well as improve the quality of life, shorten the length ofhospitalisation, and reduce the cost of patients forsurgery. Whether DEB-TACE can prolong life and improve life quality in other solid tumors is further evaluated. Yonghua et al. have treated oesophageal cancer patients with DEB-TACE, respectively [74]. The above study can fully confirm that DEB-TACE not only prolongs a patient's survival time but also has more advantages than c-TACE in improving patients' quality of life after surgery. The current study found that DEB-TACE is effective in controlling the accompanying symptoms of tumors and improving the quality of life for patients with various tumors. To provide an objective theoretical basis, DEB has been used to embolize the renal arteries of pigs in a 24-case porcine renal model in animal experiments, and then the infarction extent of the renal tissues, renal function, and other indicators are observed; the results have confirmed a good infarction effect, with no renal function damage found, indicating that it is safe. Subsequently, some scholars have carried out clinical studies on this basis [75]. Based on the above study, it can be initially concluded that DEB-TACE can not only prolong the survival time of patients but also is more significantly advantageous in promoting tumor necrosis and alleviating the accompanying symptoms of tumors; it further confirms that DEB-TACE does not lose the physiological functions of the original organs. Despite the DEB-TACE's ability to treat visceral solid tumors, it can also benefit solid tumors in superficial organs. As has been shown by a retrospective analysis of 15 patients with locally advanced breast cancer partial remission rates of 60%, 73.3%, and 73.7% were achieved at 1, 3, and 5 months after DEB-TACE treatment, respectively, and that there is a significant decrease in the breast cancer-specific tumor marker CA-153 [76]. In the above studies, various solid tumors were involved, such as visceral solid tumors and superficial organ solid tumors; DEB-TACE can makethe tumors shrink to a certain extent, improve patient’s quality of life, and prolong patient's survival time, and it has a good short-term efficacy. In the future, more studies should be conducted to deeply explore the effectiveness of DEB-TACE in treating different types of tumors. Ultimately, we can load other chemotherapeutic agents for DEB-TACE treatment for patients with various tumors.

## Adverse effects caused by callisperes^®^ drug-eluting bead transarterial chemoembolisation in the treatment of solid tumors

DEB-TACE, while significantly improving patient survival, quality of life, and symptom management in comparison to conventional chemotherapy, is not devoid of adverse effects. This section discusses about the common adverse reactions associated with DEB-TACE, their etiologies, and management approaches.

### Fever

Fever is one of the most common adverse reactions of DEB-TACE. Jiao Zhang et al. have shown that the occurrence of fever after DEB-TACE was as high as 31% [77].The etiology of postoperative fever can be multifactorial, including (1) excessive embolization due to overdosage of DEB; (2) due to the large size of the tumor, DEB-TACE results in a large area of tumor infarction [78]; (3) DEB enters the blood vessel and is embedded in the target vessel, thus blocking the tumor’s blood supply, causing tumor ischemia and hypoxia, stimulating the production of cell death factors,causing the secretion of interleukin-1, tumor necrosis factor, and other pyrogens [79], which act on the thermoregulatory centers, leading to elevation of the regulatory point and increased heat production, thereby causing fever. In the study of Tan-Yang Zhou et al., 88 patients with intrahepatic cholangiocarcinoma have undergone a total of 126 DEB-TACE surgeries, and 46.0% of the patients show elevated body temperatures after treatment; however, all of them are hypofebrile, and their body temperatures return to normal after symptomatic therapies [80]. Meanwhile, in a study by Jinpeng Li et al., 65.12% of patients exhibit elevated temperatures, of whom about 2/3 were hypothermic [81]. Additionally, in a recent meta-analysis of 16 retrospective studies, for 722 patients with DEB-TACE treatment and 732 patients with c-TACE treatment, there is no statistically significant difference in the proportion of patients who develop fever in the two groups after surgery [82]. Studies above have demonstrated the increasing postoperative temperature is common after DEB-TACE treatment, but mainly patients have low fever, and very few patients experience high fever. According to the mechanism of postoperative fever in patients, low fever after surgery indicates that the tumor necrosis is more significant factor.For this reason, we can inform the patients before the operation that there may getfever symptoms after the operation to avoid the patients' postoperative tension and anxiety that may increase their psychological burden and affect their quality of life.

### Pain

Pain following DEB-TACE results from microsphere-induced arterial embolization, leading to tissue ischemia and hypoxia. on the one hand, when tissues face hypoxia, acidic pain-causing products will be generated through anaerobic fermentation. On the other hand, hypoxia will promote the release of prostaglandins, which activate mast cells through the signaling pathway and aggravate the aseptic inflammation response [83], and ultimately act on the visceral receptors, reaching the central nerves through nerve transmission, resulting in the pain in the ischemic region [84]. Zhiheng Wang et al. have reported a study of DEB-TACE for breast cancer; it found that all the participants experience pain after surgery, and 20% of the patients have moderate pain as determined by the visual analogue scoring criteria [76]. This result reflects the commonness of postoperative pain after DEB-TACE on the one hand. On the other hand, the study's sample size was only six patients, and there may be possible bias caused by the small sample size. Additionally, a meta-analysis comparing DEB-TACE and c-TACE treatments shows that the postoperative pain after DEB-TACE treatment is not significantly different from that of c-TACE treatment. However, the authors concluded that there is bias in the data by Egger's test [61]. Although DEB-TACE and c-TACE have different drug types and dosages, some procedures are similar, and thus the incidence of postoperative pain is approximately the same. Therefore, to further clarify the incidence of postoperative pain after DEB-TACE, bronchial arterial perfusion chemotherapy and DEB-BACE is compared for the treatment of lung cancer in a clinical study, it has been shown that some of the patients experience mild symptoms of chest pain, which is not statistically significant after analyses [66]. Considering that pain after previous bronchial artery perfusion chemotherapy is safe and controllable, DEB-BACE-induced chest pain may also be secure and controllable. The studies above have confirmed that pain is a common postoperative complication. Nevertheless, regarding the severity of pain, the current studies are not sufficient. On the one hand, it mainly lies in the lack of controlled analysis in the present study; on the other hand, the small sample size may also be a reason. Therefore, a larger sample size should be incorporated in the future to improve the deficiencies at this stage and provide more objective data for exploration and analysis.

### Gastrointestinal reactions

Gastrointestinal symptoms are one of the most common adverse effects of c-TACE [85]. Additionally, nausea and vomiting are found to be along with DEB-TACE [86]. The mechanism is that DEB-TACE increases the sensitivity of chemotherapeutic agents to tumor tissues, producing tumor cytotoxicity, and thus causing nausea and vomiting. A previous study has shown that chemotherapeutic agents reduce the activity of drug-metabolizing enzymes in the gastrointestinal tract, aggravating toxic side effects in the GI tract [87]. Several clinical studies have analyzed the severity of nausea and vomiting symptoms after DEB-TACE, including an exploratory clinical analysis of 172 patients with hepatocellular carcinoma treated with DEB-TACE; gastrointestinal reactions are observed in 49.42% of the patients, but all of them are mild to moderate degree, and no severe nausea or vomiting is observed [82]. In another study including 42 patients with colorectal cancer with liver metastases loaded with irinotecan for DEB-TACE, 42.8% of the patients experience nausea and vomiting; all of them are in grade 1 or 2, and no nausea or vomiting of grade 3 or higher is seen [61]. No severe GI reactions are seen in any of the above studies. Therefore, although nausea and vomiting are common adverse reactions after DEB-TACE, their degree is relatively mild. Even though the symptoms of nausea and vomiting after DEB-TACE are not severe, they inevitably cause discomfort to patients. Hence, proton pump inhibitors can be routinely used for symptomatic treatment before and after surgery, which in turn reduces the patients' postoperative gastrointestinal reactions.

## Summary, prospects and implications for future research

With the continuous exploration of scientific research and the rapid development of medical technology, the efficacy of DEB-TACE on solid tumors has been more and more clarified. DEB-TACE, due to the non-degradable biological properties of the drug-carrying microspheres, not only maintains the embolization function of the traditional arterial embolization but also has a long-lasting embolization time, which breaks the short-lived embolization of previous c-TACE and promotes the complete embolization [88]. At the same time, using microspheres as carriers for loading anti-tumor chemotherapeutic drugs could prevent the drugs from entering the circulatory system of the human body and deliver them directly to the blood vessels of the target tumors, which can reduce the metabolism and wastage of drugs, increase drug concentration around the tumor cells and tissues, enhance the therapeutic efficacy of the anti-tumor therapy, lower the systemic blood concentration, and decrease the adverse reactions of the chemotherapeutic drugs [89]. Moreover, DEB has the property of slow release of chemotherapeutic drugs, which can increase the time and space between chemotherapeutic drugs and tumor tissues, and prolong the anti-tumor effect. Although the cost of DEB-TACE is higher than systemic chemotherapy, it has entered the reimbursement catalogue of medical insurance in many regions in China. Additionally, patients treated with DEB-TACE have a shorter average hospitalization time than that those treated with systemic chemotherapy, which saves the cost of hospitalization to a certain extent. Reviewing relevant literature, we found that domestic DEB-TACE has relatively more applications in hepatocellular carcinoma to confirm its efficacy and safety.

Furthermore, the application of DEB-TACE has been gradually reported in lung cancer, oesophagal cancer, renal cancer, bladder cancer, soft tissue sarcoma, and other related solid tumors; the results of these studies also show that it can prolong the survival of patients and reduce the toxic side effects caused by chemotherapeutic drugs. The disadvantage is that the study's sample size in solid tumors other than liver cancer is relatively small. Therefore, it is necessary to further enlarge the study's sample size to confirm the efficacy and safety of DEB-TACE more strongly in treating solid tumors. Moreover, no severe complications (such as spinal cord injury and ectopic embolism) have been reported after DEB-TACE for the time being, as described in theoretical studies. The technical proficiency and mastery of the clinical operator are the determining factors in avoiding severe complications. Therefore, as clinicians, we should improve our ability to reduce the occurrence of complications.

Due to the characteristic of immune escape, tumor cells are easy to undergo recurrence and metastasis, which is one of the reasons why tumors are challenging to treat. For this reason, a single chemotherapeutic drug may not be effective in killing the tumor cells. In the future, we consider how to make various chemotherapeutic drugs adsorbed on the DEB together to enhance the anti-tumor efficacy, how to select targeted chemotherapeutic drugs for loading on the DEB for anti-tumor treatment according to different tumors, or how to formulate the precise dose of the medicine to be adsorbed on the DEB according to the patient's body surface area. The purpose of DEB-TACE is to achieve individualized anti-tumor therapy. DEB-TACE is expected to optimize its function and widely applied to solid tumors in the future.

## Data Availability

No data were available for this review.
